# Effects of Self-Concept on Narcissism: Mediational Role of Perceived Parenting

**DOI:** 10.3389/fpsyg.2021.674679

**Published:** 2021-09-30

**Authors:** Maryam Farzand, Yagmur Cerkez, Engin Baysen

**Affiliations:** Guidance and Counseling Psychology, Near East University, Nicosia, Cyprus

**Keywords:** narcissism, perceived parenting, self-concept, behavior, mediation

## Abstract

An increase in narcissism has been reported by experts over the years. Narcissists bring a lot of negative consequences to themselves and to the people around them. This study investigates that perceived parenting leads to the development of inflated, unstable self-concept. The inflated self-concept lays the framework for the development of narcissistic traits among individuals; perceived parenting affects this relationship. A sample of 628 adults was taken from North Cyprus through purposive sampling. Scales for perceived parenting, self-concept, and narcissism were administered. Results showed that the statistically significant variance in the self-concept is explained by perceived parenting. Both mothers and fathers contributed significantly to the development of inflated self-concept. Moreover, multiple linear regression results showed a higher significant variance in narcissistic traits due to self-concept compared with perceived parenting. Mediational tests also showed that high levels of narcissistic traits were indirectly related to self-concept through perceived parenting. The study findings have challenged the notion of only mothers being responsible for narcissistic traits among their children. The study results also suggest that targeting self-concept in individuals with narcissistic traits may be a good directive for therapeutic interventions.

## Introduction

The family is the prime environment for behavioral development (O'Connor and Scott, 2007). Problematic families having a lack of parental warmth and concern for children, and harsh and inconsistent disciplinary practices are identified as risk factors for the development of narcissistic traits (Ormel et al., 2005; Mahajan et al., 2010; Cutuli et al., 2012; Chang, 2014; Bushman et al., 2016). Even though most of the studies talk about the parental impact on narcissistic behaviors, however, not all the children undergoing the same parental practices develop narcissistic tendencies (Finzi-Dottan and Cohen, 2010). In this study, one of the objectives to take perceived parenting into account is that people create their own realities based on their perceptions which may or may not be factual but are real for the individual. Another objective is to find if the inflated self-concept of an individual may provide the foundation leading to the development of narcissistic traits; perceived parenting interactions between the child and the parents influence this relationship.

Primarily, perceived parenting involves how individuals perceive regular interactions that a parent had in order to nurture and discipline them including certain practices that parents engage in while raising their children such as imposing certain rules, schedules, punishments, and rewards (Dixon et al., 2008; Assadi et al., 2011). Individuals fostered in an environment of parental acceptance, dialogue, and affection have a strong self-concept (Calafat et al., 2014). In contrast, parental coercive control reduces the self-concept of an individual (Boudreault-Bouchard et al., 2013).

Self-concept refers to the totality of a complex and dynamic system of learned attitudes, opinions, and feelings that each holds to be true about the personal existence of an individual (Tan and Yates, 2007), which remains the ultimate answer to the question, “Who am I?” Contemporary self-concept research and theory assume that individuals establish self-concept in relation to different domains making self-concept a multidimensional construct such as social, competence, affect, physical, academics, family, gender roles and sexuality, racial identity, and numerous others (Bracken, 1992; Bong and Clark, 2010). Cooley (1902) used the term looking glass self to state that other important individuals especially in the early years of an individual life serve as a mirror to which children relate and perceive themselves. He stated that associated sentiments of children of appraisals of parents of them are closely impacting the self-concept than the actual appraisals. Similarly, Mead (1934) also pointed out that we distinguished ourselves by starting to think about what significant others think of us and then incorporate their perceptions into our self-concept (Seymour, 1973).

Kohut (1971, 1977) and Kernberg (1975) stated that parental excessive criticism and hostility lead to feelings of inadequacy in children and prevent positive self-regard to be developed. To deal with these feelings of inadequacy, children seek approval and admiration from others in order to inflate their self-worth. They also proposed that adult narcissism is actually deep-rooted in early childhood experiences, and interpretation of individuals of those experiences may be explained as a defensive response toward parental disapproval and lack of acceptance (Kohut, 1966; Kernberg, 1975). In contrast, Millon (1969, 1981) believed that perceptions of individuals of over-permissive parenting behaviors and excessive parental indulgence are related to the development of narcissism. Brummelman et al. (2015) claimed that children learn the behavior modeled by their parents according to their understanding and internalize these beliefs that they are superior to others and entitled to special treatment ultimately leading to excessive self-love (Akers, 2000).

Narcissism is characterized by grandiosity, arrogant, or egotistical behaviors, feelings of superiority and entitlement, and a lack of concern or empathy for others (Cain and Boussi, 2020). With high levels of narcissism to be referred to as pathological, it is otherwise considered as a normally distributed personality trait (Raskin and Hall, 1979) characterized by intrapersonal and interpersonal strategies in order to maximize and protect self-esteem, that is, the general feelings about the underlying cognitions of self-worth (Morf and Rhodewalt, 2001). With much of the research work done taking self-esteem into account with respect to narcissism, very few studies are available linking self-concept with the narcissistic tendencies. While self-concept is the cognitive or descriptive component of an individual, self-esteem is simply the evaluation of those cognitions. To improve the narcissistic traits, if the self-concept, i.e., the underlying cognitions are worked on and changed, the evaluation of these cognitions (i.e., self-esteem) will also be kept in check.

The literature mainly talks about two types of narcissism, that is, vulnerable and grandiose (Rose, 2002). Vulnerable narcissists are distinguished by having self-doubt, insecurity, profound feelings of inferiority, depression, introverts, and being oversensitive to criticism (Schoenleber et al., 2011). In contrast, grandiose narcissists are extremely assertive and extroverts with the display of dominance and grandiosity (Ng et al., 2011). The focus of this study was on grandiose narcissism and its association to inflated self-concept. Perceived parenting is argued to act as an arbitrator linking the two.

As mentioned, the self-concept of individuals is affected by many factors. One of the most influencing factors is the evaluation of other people toward the individual, especially the significant others who are mainly parents in the early years of his/her life (Peterson and Rollins, 1986). Many other researchers also suggested that parenting starts to affect from early years of life (Baumrind, 1991; Jackson-Newsom et al., 2008; Park et al., 2010). Previous studies also showed inflated self-concept to be associated with a range of mental health indicators. Emmons (1984) found that narcissistic personality inventory (NPI) scores positively correlated with self-esteem, extraversion, dominance, and independence and negatively correlated with abasement, self-ideal discrepancy, neuroticism, and social anxiety. Some degree of narcissism as measured by the NPI appears to be tapping into the benefits associated with high self-esteem and may not be maladaptive. Emmons (1984) concluded that there may be a curvilinear relationship among self-evaluation, narcissism, and adjustment.

Despite a lot of work been done in the social and personality context on narcissism, it is a complex problem that is yet to find a solution in the twenty-first century (Issurdatt, 2011; MacLaren and Best, 2013; Zhang et al., 2015; Wright et al., 2017). Elevated narcissism in adults often sets up a cascade of interpersonal and mental health challenges reinforcing the need to understand its concomitants. Experiences of maltreatment and different perceived parenting styles have been implicated (Schie et al., 2020). The previous theorizing by clinical psychologists suggests that narcissism of adults may be related to parenting practices (Kernberg, 1975; Kohut, 1977); however, a lack of consensus surrounding the conceptualization of narcissism can be found.

Horton et al. (2006) studied parenting and healthy and unhealthy narcissism in-depth and found parental warmth to be positively associated with both while psychological control of parents was positively significant only with unhealthy narcissism. The lesser the parents monitored, the higher the narcissism scores tended to be. These findings were also consistent with the study carried by Winner and Nicholson (2018). Study conducted by Watson and Clark (1992) on authoritative, permissive, and authoritarian parenting styles of Baumrind (1966) showed that perceived parental authoritativeness associates with less narcissistic maladjustment, parental permissiveness associates with immature grandiosity, and authoritarianism correlates with inadequate idealization.

Otway and Vignoles (2006) studied different psychotherapeutic theories that provide a contrasting explanation of adult narcissism resulting either from parental coldness or excessive parental admiration during the early years of life. They found that recollections of parental coldness and excessive parental admiration predicted both overt and covert narcissism. When modeled together, the effects of each were stronger than separate. Results demonstrated that the paradoxical combination of grandiosity and fragility in adult narcissism may be explained by the combination of childhood experiences. These results are also consistent with the literature backing parental overindulgence and unchecked affection leading to narcissism (Capron, 2004; Brummelman et al., 2015).

Most of the earlier studies were related to the parenting characteristics of mothers to associate with narcissism but comparatively fewer studies were done on the role of the fathers; however, that is changing gradually with studies taking father figures and their parenting impact into consideration (Nurmi and Aunola, 2005; Kakihara et al., 2010). Similarly, much research has been done on narcissism and self-esteem while less work has been done in regard to self-concept (Ryckmann, 1993; Chang et al., 2003). This study aims to posit that parenting cannot directly affect narcissistic development without impacting and developing the pertinent inflated and unstable self-concept first, which then incite narcissistic traits, the assertion made after sifting several of the studies done on narcissism and self-esteem (Watson et al., 2010; Brummelman et al., 2018; Schie et al., 2020).

## Methods

### Participants and Procedure

The purposive convenient sample of 628 adults was given the questionnaires to fill individually. Participants included 54.6% (*N* = 343) males and 45.4% (*N* = 285) females. Most of the literature reviews mentioned adolescence to be up to the age of 20 years while other studies mentioning adulthood to be starting from the age of 20 or 21 years (Furstenberg et al., 2004; Barzeva et al., 2019). Age 20 was overlapped between the two categories; therefore, after the committee approach, 21 years and older adults were approached from North Cyprus for this study. The maximum age of adults who participated in this study was 50 years. They were divided into three groups as follows: early adults (aged 21–30 years), early middle age (aged 31–40 years), and middle-age adults (aged 41–50 years). About 43.6% (*N* = 274) of the participants belonged to early adults, 34.9% (*N* = 219) belonged to early middle age, and 21.5% (*N* = 135) belonged to middle-age adults (Table 1).

**Table 1:** Demographic details of the sample (*N* = 628).

**Demographic variables**	***F***	**%**
**Age**		
21–30	274	43.6
31–40	219	34.9
41–50	135	21.5
**Gender**		
Male	343	54.6
Female	285	45.4

All the scales utilized in this study were administrated after obtaining permissions from the respective authors. Permission was also sought from the Ethics Committee Board to carry out the research. Participants were briefed about the objective of this study. After informed consent, the participants first completed the demographic information sheet along with the research scales of Alabama Parenting Questionnaire (APQ), Six Factor Self Concept Scale (SFSCS), and NPI to complete in one sitting. They were also briefed that anonymity and confidentiality will be maintained and are free to leave if they feel so. It took approximately 20–25 min for the participants to fill the scales.

### Measures

#### Perceived Parenting

Alabama Parenting Questionnaire developed by Frick (1991) is used to study perceived parenting in this study. In earlier research, the scale showed the internal consistency ranging from α = 0 0.63–0.80 (Shelton et al., 1996). It consisted of 42 items with 9 items having 2 statements for each mother and father. It is rated using a five-point Likert-type scale (1 = never, 5 = always). The scale consists of five subscales, namely, positive parenting, poor monitoring, inconsistent discipline, involvement, corporal punishment, and other discipline practices. The scores on each item were added up to get a composite perceived parenting score with a high score indicative of more of the construct. We also created composite scores for perceived parenting of mothers and fathers by summing the scores only on the items relevant to mothers and vice versa. The scale was seen to be internally consistent in this study (α = 0.75; Table 2).

**Table 2:** Descriptive statistics of Alabama Parenting Questionnaire (APQ), Narcissistic Personality Inventory, and Six Factor Self Concept Scale (SFSCS) and their subscales.

					**Score range**		
**Variables**	***N***	***M***	***SD***	**α**	**Potential**	**Actual**	**Skewness**
Perceived parenting	628	152.39	17.04	0.75	51–255	97–225	0.38
(APQ)							
Positive parenting	628	19.23	5.17	0.70	6–30	6–30	2.28
Poor monitoring	628	30.51	6.56	0.76	10–50	12–44	−0.33
Inconsistent discipline	628	15.06	5.05	0.80	6–30	6–30	0.44
Involvement	628	58.47	9.69	0.72	19–95	26–94	0.16
Corporal punishment	628	10.19	2.84	0.69	3–15	3–15	−0.41
Other disciplines	628	18.92	5.21	0.78	7–35	7–35	0.29
Self-concept (SFSCS)	628	139.85	28.59	0.83	36–252	74–244	0.27
Power	628	28.21	9.15	0.67	7–49	8–48	0.75
Task accomplishment	628	22.31	7.23	0.72	6–42	8–42	0.31
Giftedness	628	19.20	6.07	0.72	5–35	5–35	−0.05
Vulnerability	628	23.99	7.23	0.72	6–42	6–42	0.01
Likeability	628	22.09	8.63	0.81	6–42	6–41	0.27
Morality	628	24.05	9.99	0.74	6–42	6–42	1.09
Narcissism (NPI)	628	25.44	6.52	0.80	0–40	4–39	−0.43
Authority	628	5.07	2.24	0.72	0–8	0–8	−0.43
Self-sufficiency	628	3.89	2.10	0.82	0–6	0–6	−0.54
Superiority	628	3.21	1.64	0.71	0–5	0–5	−0.54
Exhibitionism	628	4.20	2.27	0.79	0–7	0–7	−0.37
Exploitativeness	628	3.08	1.71	0.74	0–5	0–5	−0.36
Vanity	628	1.83	1.15	0.70	0–3	0–3	−0.39
Entitlement	628	4.16	2.03	0.83	0–6	0–6	−0.66

#### Self-Concept

Six Factor Self Concept Scale developed by Stake (1994) is used in this study to operationalize self-concept. It consisted of 36 items with 7-point Likert scale (1 = never or almost never true of you, 7 = almost or always true of you). The scale consisted of six subscales, namely, power, task accomplishment, giftedness, vulnerability, likeability, and morality with alpha coefficients ranging between 0.76 and 0.86 (Stake, 1994; Jedouri and Rajeh, 2020). All the scores given by the participants on the items were added up to create a composite self-concept score with a high score suggesting more of the construct. The scale has an internal consistency of α = 0.85 for this study (Table 2).

#### Grandiose Narcissism

Participants filled the NPI developed by Raskin and Hall (1979) which is mostly used and effectively validated the measure of grandiose narcissism (Tamborski and Brown, 2011). It consisted of 40 items having one narcissistic and one non-narcissistic option. A single composite narcissism score is created by counting up the number of narcissistic options with higher scores indicative of more of the construct. It has seven subscales, namely, authority, self-sufficiency, superiority, exhibitionism, exploitativeness, vanity, and entitlement with a reliability coefficient ranging from 0.74 to 0.90 (Raskin and Terry, 1988; Rosario and White, 2005). The reliability coefficient in this study is 0.80 (Table 2).

### Data Analysis

Data analysis was carried out on the gathered research data using Statistical Package for the Social Sciences 25. Process version 3.5 by Andrew F. Hayes was used for mediation analysis.

## Results

### Correlations Among Study Variables

We started by checking the correlations among perceived parenting, self-concept, narcissism, and their subscales (Table 2). Perceived parenting and self-concept were found to have moderate positive correlation, *r* = 0.30, *p* < 0.01. Perceived parenting and narcissism were also found to be positively correlated, *r* = 0.23, *p* < 0.01. Importantly, involvement, *r* = 0.28, *p* < 0.01; positive parenting, *r* = 0.33, *p* < 0.01; and corporal punishment subscales of APQ, *r* = 0.28, *p* < 0.01 were found to be positively correlated with self-concept. Involvement, *r* = 0.33, *p* < 0.01; positive parenting, *r* = 0.27, *p* < 0.01; poor monitoring, *r* = 0.8, *p* < 0.05; and corporal punishment *r* = 0.10, *p* < 0.05 subscales of APQ were also found to be positively correlated with narcissism. Poor monitoring subscale of APQ negatively correlated with task accomplishment, *r* = −0.08, *p* < 0.05 and likeability subscale of SFSCS, *r* = −0.11, *p* < 0.05. Poor monitoring was also negatively correlated with the authority, *r* = −0.08, *p* < 0.05 and exploitativeness subscale of NPI, *r* = −0.10, *p* < 0.05.

Self-concept was found to be significantly correlated with narcissism, *r* = 0.51, *p* < 0.01, and all of its subscales. Most of the subscales of self-concept positively correlated with the subscales of NPI with significant correlations observed among task accomplishment subscale of SFSCS and authority subscale of NPI, *r* = 0.40, *p* < 0.01; likeability subscale of SFSCS and authority subscale of NPI, *r* = 0.69, *p* < 0.01; and likeability subscale of self-concept and exhibitionism subscale of NPI, *r* = 0.44, *p* < 0.01 (Table 3).

**Table 3:** Correlation matrix between subscales of APQ, Narcissistic Personality Inventory and its subscales, and SFSCS and its subscales (*N* = 628).

	**APQ^a^**	**Inv^b^**	**p.p^c^**	**p.m^d^**	**i.d^e^**	**c.p^f^**	**o.d.p^g^**	**NPI^h^**	**Aut^i^**	**s.s^j^**	**Sup^k^**	**Exh^l^**	**Exp^m^**	**Van^n^**	**Ent^o^**	**SFSCS^p^**	**Pow^1^**	**t.a^r^**	**Gif^s^**	**Vul^t^**	**Lik^u^**	**Mor^v^**
APQ^a^	–	0.69^**^	0.55^**^	0.39^**^	0.25^**^	0.37^**^	0.42^**^	0.23^**^	0.26^**^	0.12^*^	−0.01	0.23^**^	0.03	0.01	0.11^**^	0.30^**^	0.06	0.19^**^	0.09^*^	0.16^**^	0.29^**^	0.15^**^
Involvement		–	0.28^**^	0.03	−0.04	0.17^**^	0.11^**^	0.24^**^	0.33^**^	0.07	0.06	0.19^**^	0.06	0.02	0.08^*^	0.28^**^	0.10^**^	0.25^**^	0.04	0.11^**^	0.34^**^	0.11^**^
Positive parenting			–	0.03	−0.01	0.24^**^	0.10^**^	0.32^**^	0.27^**^	0.21^**^	−0.01	0.32^**^	0.03	0.06	0.16^**^	0.33^**^	0.11^*^	0.19^**^	0.16^**^	0.07	0.38^**^	0.22^**^
Poor monitoring				–	−0.12^*^	0.06	−0.07	−0.06	−0.08^*^	0.01	0.01	−0.03	−0.10^*^	−0.2	0.01	−0.04	0.02	−0.08^*^	0.03	0.05	−0.11^*^	−0.03
Inconsistent discipline					–	−0.05	0.10^*^	0.05	−0.02	0.04	−0.01	0.08^*^	0.07	0.01	0.02	0.03	−0.03	0.06	−0.01	0.07	0.04	−0.01
Corporal punishment						–	0.09^*^	0.09^*^	0.17^**^	0.04	−0.03	0.14^**^	−0.01	0.02	−0.03	0.11^**^	0.02	0.02	0.2	0.06	0.15^**^	0.11^**^
Other discipline practices							–	−0.01	0.04	−0.02	−0.13^*^	−0.04	0.03	−0.01	0.05	−0.01	−0.09^*^	0.04	−0.02	0.05^*^	0.02	0.01
NPI^h^								–	0.54^**^	0.47^**^	0.43^**^	0.63^**^	0.49^**^	0.36^**^	0.48^**^	0.51^**^	0.24^**^	0.40^**^	0.19^**^	0.14^**^	0.55^**^	0.25^**^
Authority									–	0.20^**^	0.06	0.15^**^	0.14^**^	0.10^**^	0.06	0.51^**^	0.08^*^	0.40^**^	0.12^**^	0.18^**^	0.69^**^	0.27^**^
Self sufficiency										–	0.02	0.14^**^	0.16^*^	0.11^*^	0.08^*^	0.24^**^	0.10^*^	0.14^*^	0.17^**^	0.10^**^	0.25^**^	0.10^*^
Superiority											–	0.18^**^	0.13^**^	0.08^*^	0.11^**^	0.22^**^	0.27^**^	0.18^**^	−0.03^*^	0.03^*^	0.17^**^	0.09^*^
Exhibitionism												–	0.17^**^	0.17^**^	0.21^**^	0.41^**^	0.16^**^	0.33^**^	0.21^**^	0.13^**^	0.44^**^	0.17^**^
Exploitativeness													–	0.13^**^	0.13^**^	0.12^**^	−0.01	0.14^**^	0.05	−0.04	0.13^**^	0.13^**^
Vanity														–	0.04	0.14^**^	0.04	0.13^**^	0.11^**^	0.04	0.06	0.11^**^
Entitlement															–	0.12^**^	0.18^**^	0.09^*^	0.04	0.01	0.09^*^	0.01
SFSCS^p^																–	0.49^**^	0.73^**^	0.40^**^	0.45^**^	0.75^**^	0.66^**^
Power																	–	0.21^**^	0.02	0.07	0.19^**^	0.11^**^
Task accomplishment																		–	0.17^**^	0.22^**^	0.51^**^	0.46^**^
Giftedness																			–	0.06	0.24^**^	0.13^*^
Vulnerability																				–	0.23^**^	0.08^*^
Likeability																					–	0.40^**^
Morality																						–

a *Alabama Parenting Questionnaire.*

b *Involvement.*

c *Positive parenting.*

d *Poor monitoring.*

e *Inconsistent discipline.*

f *Corporal punishment.*

g *Other discipline practices.*

h *Narcissistic Personality Inventory.*

i *Authority.*

j *Self-sufficiency.*

k *Superiority.*

l *Exhibitionism.*

m *Exploitativeness.*

n *Vanity.*

o *Entitlement.*

p *Six Factor Self Concept Scale.*

q *Power.*

r *Task accomplishment.*

s *Giftedness.*

t *Vulnerability.*

u *Likeability.*

v *Morality.*

** *p <0.01 and*

* *p <0.05*.

### Age and Gender Differences on Perceived Parenting, Self-Concept, and Narcissism

To find gender differences in APQ, SFSCS, and NPI, an independent sample *t*-test was conducted. Significant gender differences were only present for NPI (*t* = 0.06, *p* < 0.05; *d* = 0.02), though the effect size was not high (Table 4), with males scoring (*M* = 65.46, *S* = 6.87) slightly higher than females (*M* = 65.42, *S* = 6.08).

**Table 4:** Gender differences on parenting, self-concept, and narcissism (*N* = 628).

	**Male (*****n*** **= 343)**		**Female (*****n*** **= 285)**				**95%** ***CI***		
**Variables**	***M***	***SD***	***M***	***SD***	***t*_**(628)**_**	***p***	***LL***	***UL***	**Cohen's *d***
APQ	151.27	16.17	151.53	16.45	−0.20	0.82	−2.83	2.30	0.02
SFSCS	138.26	28.17	140.70	26.93	−1.11	0.22	−6.79	1.90	0.09
NPI	65.46	6.87	65.42	6.08	0.06	0.04	−0.98	1.05	0.01

A one-way ANOVA (Hair et al., 2010) was conducted to compare the effects of age (early adulthood, early middle age, and late middle age) on perceived parenting, self-concept, and narcissism. An analysis of variance showed that the effect of age was significant on APQ, *F*_(2, 625)_ = 10.66, *p* < 0.001, SFSCS, *F*_(2, 625)_ = 60.87, *p* < 0.001, and NPI, *F*_(2, 625)_ = 33.63, *p* < 0.05. Furthermore, a *post-hoc* test using Bonferroni for multiple comparisons to unequal group sizes revealed that participants from late middle age (41–50 years) scored high on APQ and SFSCS. Early middle age (31–40 years) scored slightly higher than late middle age on NPI while participants from early adulthood (21–30 years) scored lower than early and late middle age on all the three scales (Table 5). As age and gender differences are seen to possibly influence narcissism, they are included in the regression analysis.

**Table 5:** Analysis of variance of adult's age (early adulthood: 21–30 years old, early middle age: 31–40 years old, and late middle age: 41–50 years old) on perceived parenting, self-concept, and narcissism (*N* = 628).

								**95%** ***CI***	
**Variables**		***M***	**SD**	***F***	***i*^1^, *j*^2^, *k*^3^**	***Mean Diff. (i*^1^, *j*^2^, *k^3^)***	**SE**	**LB**	**UB**
APQ	Early adulthood (*n* = 274)	148.28	16.45	10.66^***^	Early adulthood < early middle age^*^	*−4.35* ^*^	1.45	−7.84	−0.86
	Early middle age (*n* = 219)	152.63	16.67		NS	NS	NS	−7.28	1.15
	Late middle age (*n* = 135)	155.69	14.01		Late middle age > early adulthood^*^	7.41^*^	1.69	3.36	11.46
SFSCS	Early adulthood (*n* = 274)	128.04	29.05	60.87^***^	Early adulthood < early middle age^*^	−14.86^*^	2.29	−20.37	−9.35
	Early middle age (*n* = 219)	142.90	22.92		Early middle age < late middle age^*^	−13.70^*^	2.77	−20.35	−7.06
	Late middle age (*n* = 135)	156.61	20.33		Late middle age > early adulthood^*^	28.56^*^	2.66	22.17	34.95
NPI	Early adulthood (*n* = 274)	63.14	6.86	33.63^***^	Early adulthood < early middle age^*^	−4.10^*^	0.56	−5.45	−2.75
	Early middle age (*n* = 219)	67.23	5.721		NS	NS	NS	−1.62	1.64
	Late middle age (*n* = 135)	67.22	5.52		Late middle age > early adulthood^*^	4.09^*^	0.65	2.52	5.65

1 *number of early adults,*

2 *number of early middle age,*

3 *number of late middle age.*

* *p <0.05 and*

*** *p <0.001*.

### Perceived Parenting of Mothers and Fathers, Self-Concept, and Narcissism

To determine if mothers and fathers have any influence on the self-concept and narcissism, regression results showed that 10% statistically significant variance in the self-concept is explained by perceived parenting of mothers while 5% statistically significant variance in the self-concept is explained by perceived parenting of fathers (Table 6). In contrast, the value of adjusted *R*^2^ shows that 8% statistically significant variance in narcissism is explained by perceived parenting of mothers while 4% statistically significant variance in narcissism is explained by perceived parenting of fathers (Table 7).

**Table 6:** Linear regression analysis of perceived parenting of mothers and fathers on self-concept (*N* = 628).

**Variables**	***t***	**B**	**β**	***F***	**Adj. *R*^**2**^**
Intercept	13.99^***^	88.01		68.62^***^	0.10
Mother	8.28^***^	1.86	0.31		
Intercept	15.79^***^	101.01		37.04^***^	0.05
Father	6.09^***^	1.41	0.24		

*The dependent variable for regression is self-concept.*

*** *p <0.001*.

**Table 7:** Linear regression analysis of perceived parenting of mothers and fathers on narcissism (*N* = 628).

**Variables**	***t***	**B**	**β**	***F***	**Adj. *R*^**2**^**
Intercept	36.48^***^	54.81		51.54^***^	0.08
Mother	7.18^***^	0.39	0.28		
Intercept	38.03^***^	57.90		25.24^***^	0.04
Father	5.02^***^	0.28	0.20		

*The dependent variable for regression is narcissism.*

*** *p <0.001*.

### Mediational Role of Perceived Parenting

The consistent finding in much of the narcissism literature is that both age and gender affect narcissism (Wilson and Sibley, 2011; Grijalva et al., 2015; Berenson et al., 2017; Hoertel et al., 2018); therefore, to determine the mediating role of perceived parenting between self-concept and narcissism, both age and gender were controlled. To check the role of perceived parenting (mediator) between self-concept (independent variable) and narcissism (dependent variable), four steps devised by Baron and Kenny (1986) were followed for each mediation. These include (a) significant association between the independent variable and dependent variable, which can be mediated by a third variable, (b) significant association between independent variable and mediator, (c) significant association between the mediator and dependent variable, and (d) significant decline in independent variable and association of dependent variable when the mediator is added to the model after controlling the independent variable. For this, simple linear regression was done from the independent to dependent variable, from independent to mediator variable, and from mediator to dependent variable; then mediation is conducted. Finally, to build in confidence further on mediation findings, the *Sobel* test value was also calculated as advocated by MacKinnon et al. (1995).

**Figure 1:**
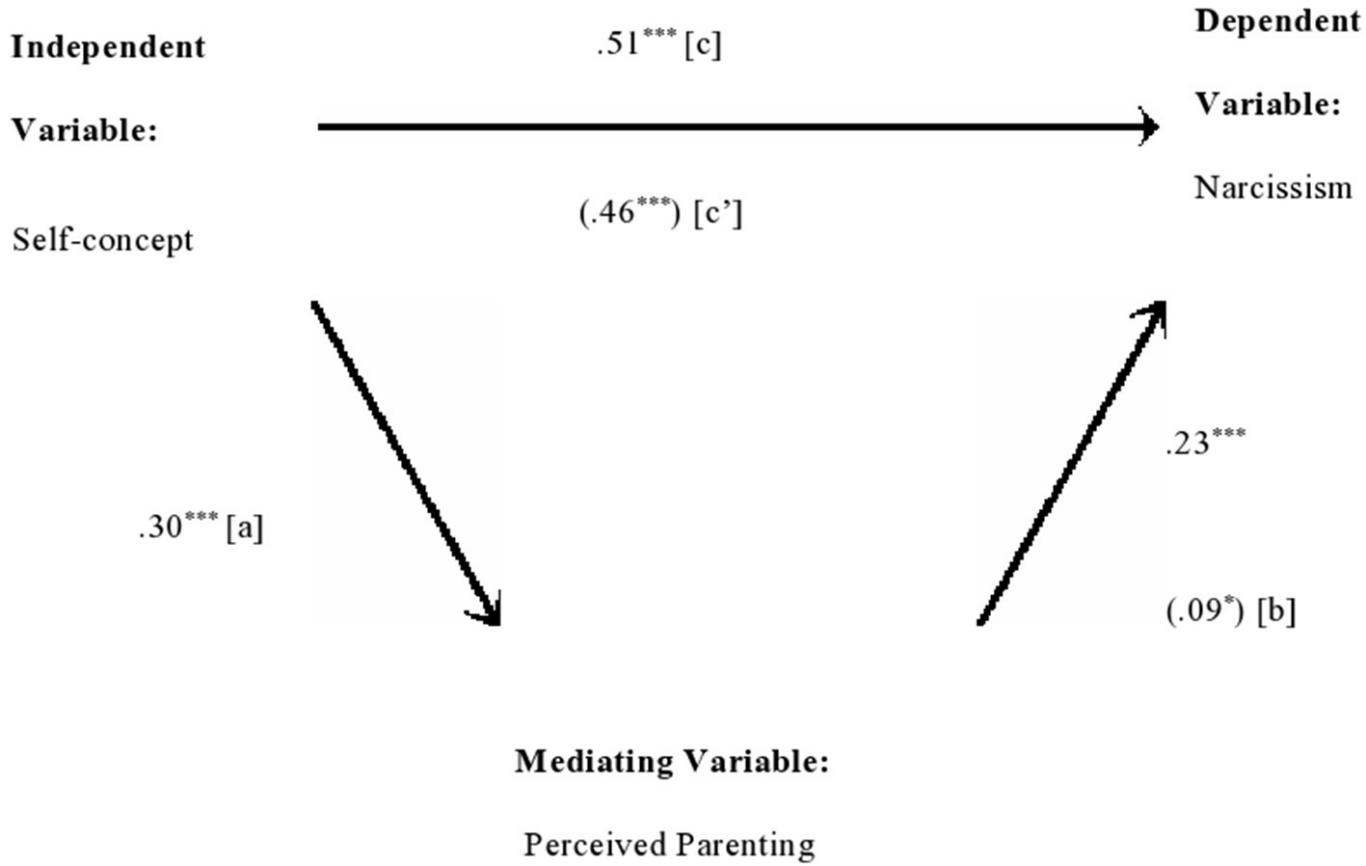
Medgraph shows indirect effect of self-concept and narcissism through perceived parenting. The numerical values in parentheses are beta weights taken from the second regression and the other values are zero order correlations. ***p < 0.001 and *p < 0.05.

Perceived parenting significantly mediates the direct relationship between self-concept and narcissism explaining about 28% variance (Table 8). The direct effects from self-concept to perceived parenting (*b* = 0.14, *SE* = 0.02, *p* < 0.001), from perceived parenting to narcissism (*b* = 0.04, *SE* = 0.01, *p* < 0.05), and from self-concept to narcissism (*b* = 0.11, *SE* = 0.01, *p* < 0.001) were positively significant (Figure 1). The indirect effect, tested using non-parametric bootstrapping (0.01), is statistically significant: 95% CI = (0.001–0.01). Sobel test further validated the indirect effect of self-concept and narcissism through perceived parenting (*z* = 3.47, *p* < 0.001).

**Table 8:** Mediating effect of perceived parenting between self-concept and narcissism (*N* = 628).

	**Narcissism**			
			**Model 2**	
			**95%** ***CI***	
**Predictors**	**Model 1 B**	**B**	***LL***	***UL***
(Constant)	129.38	43.97	39.56	38.38
Self-concept	0.14^***^	0.11^***^	0.09	0.13
Perceived Parenting		0.04^**^	0.01	0.07
Age	1.83	0.62	0.02	1.23
Gender	−0.19	−0.35	−1.22	0.53
*R* ^2^	0.08	0.28		
*_*F*_*	17.47^***^	59.77^***^		
Δ*R*^2^		0.22		
Δ*F*		187.91		

*B, unstandardized regression coefficient, R^2^, explained variance; gender and age are controlled variables;*

** *p <0.01 and*

*** *p <0.001*.

### Mediational Links Between the Subscales

To identify the unique links between the subscales of perceived parenting, self-concept, and narcissism, we regressed the total APQ subscale onto every subscale of SFSCS and NPI simultaneously and after centering all predictors and outcomes. Only involvement and positive parenting subscales of APQ predicted the relationship with narcissism significantly while other subscales did not reach significance. Thus, they are not discussed further.

**Figure 2:**
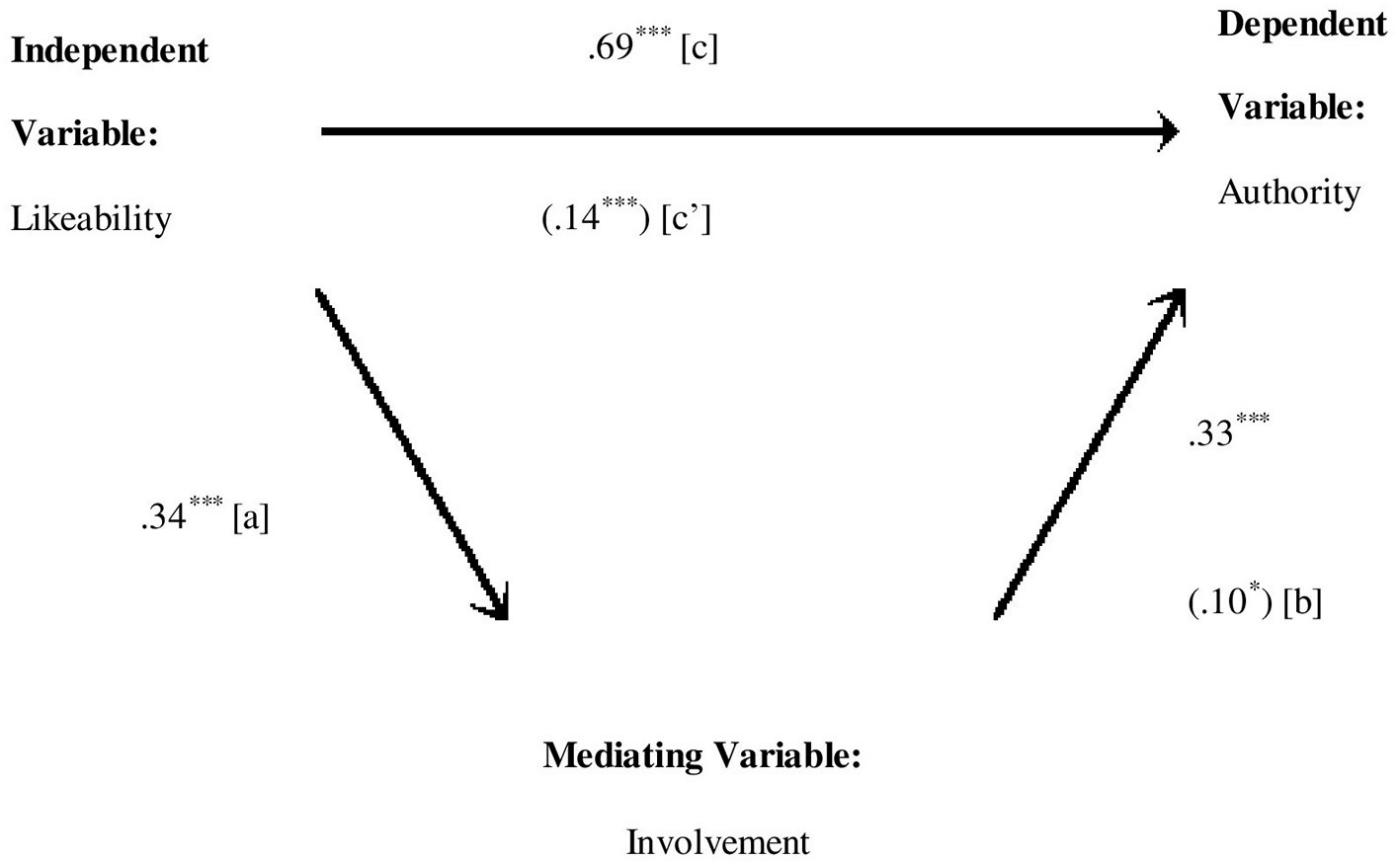
Medgraph shows indirect effect of likeability and authority through involvement (perceived). The numerical values in parentheses are beta weights taken from the second regression and the other values are zero order correlations. ***p < 0.001 and *p < 0.05.

Involvement subscale of APQ mediates the direct relationship of likeability subscale of self-concept and authority subscale of narcissism significantly explaining about 53% variance (Table 9). The direct effect from likeability to involvement (*b* = 0.29, *SE* = 0.04, *p* < 0.001), from involvement to authority (*b* = 0.03, *SE* = 0.01, *p* < 0.01), and from likeability to authority (*b* = 0.14, *SE* = 0.01, *p* < 0.001) is also positively significant (Figure 2). The indirect effect (0.01) is statistically significant: 95% CI = (0.003–0.01). Sobel test also confirmed the significant indirect effect of likeability and authority through involvement (*z* = 2.77, *p* < 0.05).

**Table 9:** Mediating effect of involvement (perceived) between likeability (self-concept) and authority (narcissism) (*N* = 628).

	**Authority**			
			**Model 2**	
			**95%** ***CI***	
**Predictors**	**Model 1 B**	**B**	***LL***	***UL***
(Constant)	39.63	7.08	6.26	7.91
Likeability	0.29^***^	0.14^***^	0.13	0.16
Involvement		0.03^**^	0.01	0.05
Age	0.29	0.57	0.38	0.75
Gender	−0.33	0.32	0.07	0.56
*R* ^2^	0.11	0.53		
*_*F*_*	26.74^***^	172.95^***^		
Δ*R*^2^		0.34		
Δ*F*		476.44^***^		

*B, unstandardized regression coefficient, R^2^, explained variance; gender and age are controlled variables;*

** *p <0.01 and*

*** *p <0.001*.

**Figure 3:**
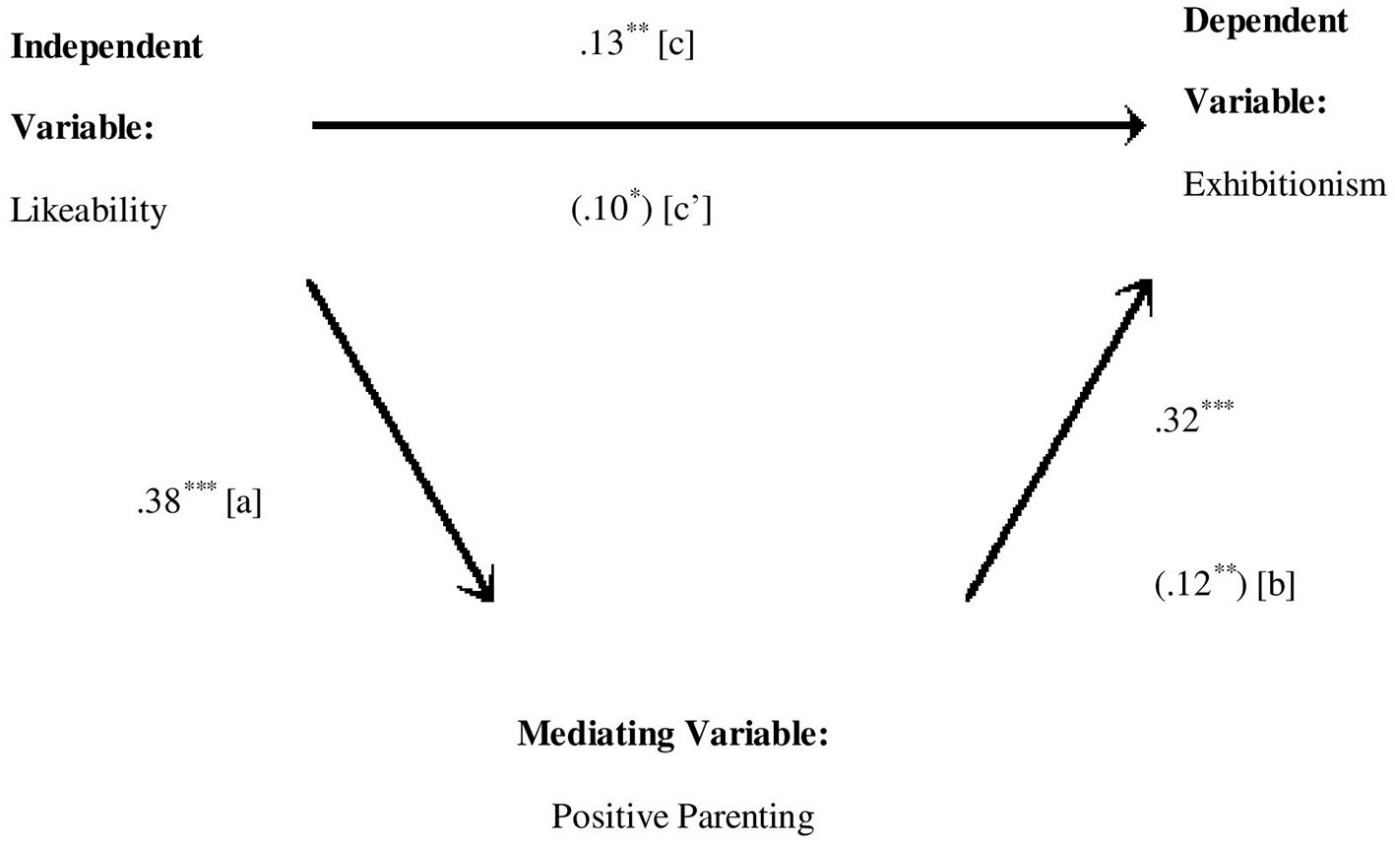
Medgraph shows indirect effect of likeability and exhibitionism through positive parenting (perceived). The numerical values in parentheses are beta weights taken from the second regression and the other values are zero order correlations. ***p < 0.001, **p < 0.01 and *p < 0.05.

Positive parenting subscale of APQ mediates the direct relationship of likeability subscale of self-concept and exhibitionism subscale of narcissism significantly explaining about 23% variance (Table 10). The direct effect from likeability to positive parenting (*b* = 0.22, *SE* = 0.02, *p* < 0.001), from positive parenting to exhibitionism (*b* = 0.09, *SE* = 0.02, *p* < 0.001) and from likeability to exhibitionism (*b* = 0.10, *SE* = 0.01, *p* < 0.001) is also positive and significant (Figure 3). The indirect effect (0.02) is statistically significant: 95% CI = (0.01–0.03). Sobel test further confirmed the significant indirect effect of likeability and authority through involvement (*z* = 4.16, *p* < 0.001).

**Table 10:** Mediating effect of positive parenting (perceived) between likeability (self-concept) and exhibitionism (narcissism) (*N* = 628).

	**Exhibitionism**			
			**Model 2**	
			**95%** ***CI***	
**Predictors**	**Model 1 B^*a*^**	**B**	***LL***	***UL***
(Constant)	14.52	8.23	7.39	9.07
Likeability	0.22^***^	0.10^***^	0.08	0.13
Positive parenting		0.09^***^	0.15	0.12
Age	−0.36	−0.08	−0.32	0.16
Gender	0.23	−0.55	−0.87	−0.24
*R* ^2 *b*^	0.15	0.23		
*_*F*_*	36.95^***^	47.43^***^		
Δ*R*^2^		0.12		
Δ*F*		94.21^***^		

a *Unstandardized regression coefficient,*

b *explained variance; gender and age are controlled variables. B, unstandardized regression coefficient, R^2^, explained variance; gender and age are controlled variables;*

** *p < 0.01 and*

*** *p < 0.001*.

## Discussion

The main aim of this study was to investigate the links between self-concept and narcissism with a focus on the mediational role of parenting. This study hypothesized that since all the children in a family subjected to the same parenting do not end up developing narcissistic traits, the development of relevant inflated self-concept leads to narcissism in individuals, and perceived parenting mediate that relationship. Bivariate correlations were first computed to determine the associations between the study variables. The results of this study were consistent with previous studies but also propounded on the mediational link between perceived parenting and narcissism. Total APQ was associated with self-concept and all its subscales except the power subscale. Involvement and positive parenting subscales of APQ showed significant association with the power subscale of SFSCS. This is consistent with the research finding of Clarke et al. (2004) that over-involvement and excessive pampering by parents result in child to become spoiled; they allow excessive freedom and flexibility in a soft structured environment to their children, in which parents abstain from surveilling the behavior of their children; they enforce no rules, regulations, or boundaries, which gives children too much freedom and power that they feel entitled to exercise even in later years (Clarke et al., 2004; Mueller, 2011). Parental monitoring was negatively associated with task accomplishment and likeability subscale of SFSCS. Even though this finding is in disagreement with some of the literature findings, Stattin and Kerr (2000) posited that excess parental monitoring is sometimes perceived as excessive control and intrusion by the children which makes it less effective; inconsistent parental monitoring affects self-efficacy, which indicate similar internal structures as self-concept (Bong and Clark, 2010).

Perceived parenting was also associated with narcissism and four of its components, namely, authority, self-sufficiency, exhibitionism, and entitlement. Parental monitoring was negatively associated with authority and exhibitionism of NPI which is consistent with the literature (Wetzel and Robins, 2016), while total SFSCS was significantly associated with the total NPI, and all of its subscale strengthening the hypothesized link between self-concept and narcissism. These findings again suggest that without setting appropriate boundaries, over affection enables narcissistic traits to develop. We left open the debate of whether these findings are due to the social learning theory by Millon (1969) that children develop narcissistic traits because their parents consider them more special and entitled than the rest and treat accordingly or parents fail to inculcate favorable self-regard in childhood that develops from positive mirroring (perspective of Kohut). Given that the data of this study are from early to middle-age adults, the perspective of Kohut emphasizes early childhood experiences, and the results of this study seem to be parallel with the approach of Millon. To date, the compiled research also suggests that the levels of parental monitoring and inconsistent parental practices are associated with the involvement of an individual in a range of antisocial and delinquent behaviors, narcissistic tendencies, and other personality disorders (Patterson, 1996; Dishion and McMahon, 1998; Crouter and Head, 2002). Perceived parental warmth was positively associated, and monitoring was negatively associated with both types of narcissism (Horton et al., 2006).

Existing studies on grandiose narcissism among adolescents and adults are closely related to their theoretical descriptions that narcissism, despite having some self-beneficiary aspects, has socially undesirable facets as well. Several studies done on children and adolescents have also shown that the overuse of positive reinforcement by parents may develop superiority and grandiose tendencies in children as they start to perceive themselves to be worthy of rewards and attention from others. Similar to this study, these findings are also consistent with the social learning theory by Millon that excessive praise and reward from parents strengthen entitlement and superiority in children. Alternatively, youth with grandiose ideation about self may further elicit positive parental attention and praise (Mechanic and Barry, 2015). In a longitudinal study done by Wetzel and Robins (2016), cross-lagged relations among parental warmth, hostility and monitoring with superiority, and exploitativeness elements of narcissism were examined among adolescents. High exploitativeness level was associated with high parental hostility, whereas lower exploitativeness level was associated with low parental monitoring while none of the parenting dimensions was related to superiority. Another study done by Coppola et al. (2020) found that narcissism of both parents was positively associated with overvaluation and the narcissistic traits of children; an indirect link between the narcissistic traits of fathers and children was partially mediated by overvaluation. Positive parenting of mothers was found to have a direct positive relation to the self-esteem of the children.

While much work has been done on the association between gender and narcissism, this study also confirmed similar results (Tschanz et al., 1998; Zhou et al., 2012; Green et al., 2020). Significant age differences were present for perceived parenting, self-concept, and narcissism. Differences by age were previously found in the parenting style perception of adolescents and maladjustments of school as studied by Jaureguizar et al. (2018). Age also seemed to impact the formation of self-concept in the work done by Marsh (1989), which stated that self-concept declines from early preadolescence to middle adolescence and then increases through early adulthood (Marsh, 1989). The influence of age is also reflected on self-concept in some other studies (McCrae and Costa, 1988; Diehl and Hay, 2011). The review of previous studies gives varied results relating to the impact of age on narcissism. Some researchers claimed that traits of narcissists such as authority and independence decline with age (Danko et al., 2009) while Peruchon (2004) associated old age with increased narcissism arguing that narcissistic tendencies in old age may help in forming friendships and gain the social support needed (Jonason and Schmitt, 2012), and offsetting isolation or other distress backing the findings of this study shows significant age differences, with older adults having more narcissistic traits.

Then, we conducted regression analyses to determine the predictive value of both mothers and fathers on self-concept and narcissism. While much of the earlier investigations emphasized the role of mothers with stronger associations of perceived maternal parenting, some studies in recent years began to highlight the importance of the role of fathers as well (Brummelman et al., 2015; Huxley and Bizumic, 2017). Results of this study showed that both mothers and fathers predict the development of self-concept and narcissism, with mothers contributing more variance to self-concept and narcissism than fathers, which is in line with the previous work (Watson at al., 1992; Huxley and Bizumic, 2017).

There was a significant mediating effect of perceived parenting in the association between self-concept and narcissism, indicating that parenting may affect the formation of self-concept which further enhances their narcissistic traits. The mediation models suggested that the involvement subscale of APQ mediated the relationship between the likeability subscale of SFSCS and the authority subscale of NPI while the positive parenting subscale of APQ mediated the relationship between likeability of SFSCS and exhibitionism subscale of NPI. Both direct and indirect effects were positively significant. These results are parallel to the study by Pesu et al. (2016) which states that the self-concept of children is mainly derived from their parents and the beliefs of the significant others. Scientific study done by Schie et al. (2020) also advocated similar results.

Despite the limitation of cross-sectional data which may produce model fit estimates that can be overestimates, underestimates, or at best only approximate estimates of fit, the work contributes to the growing literature on parenting, self-concept, and narcissism. Longitudinal studies in the future can give us estimates in the context of proper temporal ordering of variables. Also, this research relied on retrospective accounts of perceived parenting, a common practice in the literature (Watson et al., 1992; Horton et al., 2006; Otway and Vignoles, 2006), but there is a likelihood of inadvertent errors in memory recollection. However, it is encouraging that the findings of this study are parallel to the results of studies that solicited accounts of current parenting (Horton et al., 2006; Wetzel and Robins, 2016).

## Conclusion

We hope that this research paves the way for further empirical evidence along the current lines to improve the understanding of how parenting affects the development of maladaptive self-concept leading to narcissistic traits and effective parenting strategies to promote the development of healthy self-concept before unchecked narcissism may dwell into a society driven by selfishness and lacking empathy.

## Ethics Statement

The studies involving human participants were reviewed and approved by Near East University Ethics Board. The patients/participants provided their written informed consent to participate in this study.

## Author Contributions

MF was mainly responsible for the study conceptualization, data collection, and data preparation. YC was involved in report writing and EB with the data analysis. All authors contributed to the article and approved the submitted version.

## Conflict of Interest

The authors declare that the research was conducted in the absence of any commercial or financial relationships that could be construed as a potential conflict of interest.

## Publisher's Note

All claims expressed in this article are solely those of the authors and do not necessarily represent those of their affiliated organizations, or those of the publisher, the editors and the reviewers. Any product that may be evaluated in this article, or claim that may be made by its manufacturer, is not guaranteed or endorsed by the publisher.
